# The Role of AI-Based Software BrainScan in the Interpretation of Non-Contrast Head CT in Acute Ischemic Stroke: An External Validation Study

**DOI:** 10.3390/neurolint18060100

**Published:** 2026-05-22

**Authors:** Eray Halil, Ivan Sitnikov, Neli Atanasova, Petra Popova, Kostadin Kostadinov, Fares Ezeldin, Penka Atanassova

**Affiliations:** 1 Department of Neurology, Medical University of Plovdiv, 15A Vasil Aprilov Blvd., 4002 Plovdiv, Bulgaria; 2 Clinic of Neurology, UMHAT “St George”, 66 Peshtersko Shose Blvd., 4001 Plovdiv, Bulgaria; 3 Department of Radiology, UMHAT “St George”, 66 Peshtersko Shose Blvd., 4001 Plovdiv, Bulgaria; 4 Department of Social Medicine and Public Health, Medical University of Plovdiv, 15A Vasil Aprilov Blvd., 4002 Plovdiv, Bulgaria; 5 Environmental Health Division, Research Institute, Medical University of Plovdiv, 15A Vasil Aprilov Blvd., 4002 Plovdiv, Bulgaria; 6 Department of Forensic Medicine and Deontology, Medical University of Plovdiv, 15A Vasil Aprilov Blvd., 4002 Plovdiv, Bulgaria

**Keywords:** artificial intelligence, BrainScan, neuroimaging AI, ischemic stroke, deep learning, stroke detection, AI validation, computed tomography

## Abstract

Background/Objectives: Artificial intelligence (AI) tools are increasingly integrated into acute stroke imaging workflows, but real-world performance for ischemia detection on non-contrast CT (NCCT) remains incompletely validated by investigators independent of the developer. This study externally validated the BrainScan AI system in an unselected, consecutively enrolled emergency cohort. Methods: Consecutive adult patients undergoing NCCT under the routine acute stroke protocol at a single tertiary centre between January and December 2025 were prospectively enrolled. The reference standard was the post-consensus radiological diagnosis, supplemented where available by follow-up imaging and clinical course. Primary outcomes were diagnostic accuracy for ischemia and intracranial haemorrhage detection, assessed by sensitivity, specificity, predictive values, likelihood ratios, and area under the ROC curve (AUC; DeLong). Pre-specified secondary analyses included regional sensitivity, confidence-score behaviour, artefact robustness, threshold sensitivity, a cluster-robust bootstrap for within-patient correlation, and a quantitative bias analysis under non-differential reference-standard misclassification. Sample size adequacy was assessed using a precision-based framework. Results: A total of 1419 NCCT examinations from 1260 patients were analysed. Ischemia sensitivity was 59.2% (95% CI 52.1–66.1) and specificity was 99.8% (99.4–100), with an AUC of 0.930 (0.906–0.954). The Youden-optimal threshold (0.055) recovered sensitivity to 86.1% with negligible specificity loss, reflecting a markedly bimodal score distribution. Regional sensitivity was lower in infratentorial structures. Bias-corrected estimates were stable across all reference-standard parameters consistent with the data. Haemorrhage detection performed substantially better (sensitivity 96.7%; AUC 0.983). Conclusions: The system shows excellent specificity and strong discrimination but moderate sensitivity for ischemia, supporting its role as a rule-in adjunct rather than a stand-alone tool, pending multicentre validation and site-specific threshold recalibration.

## 1. Introduction

Stroke is a leading cause of death and long-term disability worldwide. Each year, over 12 million people suffer a first-time stroke and about 6.5 million die as a result [1]. The vast majority of strokes are ischemic, caused by an abrupt blockage of a cerebral artery, whereas the remaining cases are hemorrhagic due to intracranial bleeding [2]. In an ischemic stroke, the loss of blood flow leads to rapid brain injury, making stroke a true medical emergency. Fast reperfusion treatments (like intravenous thrombolysis or mechanical thrombectomy) can save brain tissue; therefore, rapid diagnosis is critical. This urgency has even given rise to the mantra “time is brain,” understanding that every minute of delay in treatment can lead to irreversible loss of neurons. Effective acute management therefore hinges on prompt recognition of stroke and determination of its type, which relies heavily on brain imaging.

### 1.1. Role of Non-Contrast CT in Acute Stroke Diagnosis

Non-contrast computed tomography (NCCT) of the brain remains the first-line imaging modality in suspected acute stroke [3]. NCCT is fast, widely available and highly sensitive for acute intracranial hemorrhage, which is its primary role—to quickly rule out hemorrhagic stroke that would contraindicate thrombolytic therapy [4]. In the hyperacute phase of ischemic stroke (within the first hours), NCCT often appears normal or shows only subtle changes because its sensitivity to early ischemic injury is limited. Up to one-third of acute ischemic strokes may not display a clear hypodense area on NCCT during the initial hours [5]. Nevertheless, trained radiologists can sometimes recognize early ischemic signs on NCCT, including loss of gray-white matter differentiation, insular “ribbon” sign, obscuration of the lentiform nucleus, and hyperdense vessel signs [6]. Despite its limitations, NCCT remains the standard first-line imaging modality in the acute evaluation of suspected stroke because of its rapid availability, broad accessibility, and ability to exclude intracranial haemorrhage.

### 1.2. AI-Based Software for Stroke Imaging Interpretation

In recent years, artificial intelligence (AI) techniques have been applied to stroke imaging to assist clinicians in faster and more accurate interpretation. Recent large-scale evaluations have highlighted the rapid expansion of AI applications in radiology and neuroimaging, particularly in acute stroke workflows [7]. Given the time-critical nature of stroke treatment, there is intense interest in leveraging AI; indeed, stroke is one of the most active areas in radiology AI research. In fact, out of 214 medical imaging AI applications approved by regulators (FDA and/or CE) to date, 73 are related to neuroimaging, and over half of those focus on stroke assessment [8]. The rationale is that AI-driven tools can analyze brain scans in seconds, potentially alerting physicians to critical findings even before a radiologist has reviewed the images. A number of AI-based decision support software platforms are now commercially available and integrated into stroke workflows [9]. These tools can perform a suite of specialized tasks across different imaging modalities: for example, automatically detecting an intracerebral hemorrhage on NCCT, identifying a large vessel occlusion (LVO) on CT angiography, quantifying early ischemic changes on NCCT via an ASPECTS calculation, and processing CT perfusion maps to delineate the ischemic core and penumbra. High-profile software packages (such as RapidAI, Viz.ai, e-ASPECTS, among others) have demonstrated the ability to flag critical findings with high speed and accuracy. For instance, one FDA-cleared AI system (CINA^®^ by Avicenna.AI) was shown to detect intracerebral hemorrhages on NCCT with ~95% accuracy, identify LVOs on CTA with ~86% accuracy, and automatically score ASPECTS within a few minutes [9,10]. In a systematic review of AI applications for ischemic stroke diagnosis and LVO identification, Murray et al. summarized the heterogeneity of machine learning and deep learning strategies used across the literature and highlighted that convolutional neural networks are commonly applied to LVO detection, while other machine-learning approaches have been used in automated assessment tasks such as ASPECTS. Importantly, the authors emphasized that reported performance metrics and evaluation standards vary substantially between studies, limiting direct comparisons and underscoring the need for standardized validation frameworks [11]. Despite these promising results, the heterogeneity of algorithms, training datasets, and validation methodologies across studies raises concerns regarding generalizability and real-world clinical performance. Recent studies have demonstrated that externally validated performance often differs substantially from internally reported metrics, underscoring the importance of real-world evaluation [12]. Consequently, independent external validation studies in routine clinical settings are essential to determine the true added value and reliability of AI-based software in acute stroke imaging.

### 1.3. Limitations of AI in Stroke Imaging

Despite encouraging results, the clinical use of AI-based stroke imaging tools remains subject to important limitations. Several studies have shown that the performance of AI algorithms may vary across different populations, scanners, and imaging protocols, raising concerns about generalizability beyond the original training environment [13,14]. This variability across settings is consistent with prior work demonstrating the sensitivity of AI performance to differences in data acquisition, population characteristics, and institutional workflows [15]. In addition, AI systems may demonstrate reduced sensitivity for small infarcts, atypical stroke presentations, and scans affected by motion or beam-hardening artifacts, which remain challenging even for expert human readers [11,16]. Another important limitation is the limited interpretability of many AI models, which may affect clinician trust and slow clinical adoption [17]. A recent systematic review and meta-analysis evaluating artificial intelligence–based models for detecting acute ischemic stroke on non-contrast CT demonstrated encouraging diagnostic performance across multiple studies. However, the authors reported substantial heterogeneity in algorithms, datasets, and evaluation metrics, and highlighted that most published models rely primarily on internal validation [14]. The limited availability of independent external validation raises concerns regarding the generalizability and real-world clinical performance of AI-based stroke imaging tools, underscoring the need for validation studies in routine clinical settings.

Within this broader landscape of incomplete external evidence, published evaluations of the BrainScan platform specifically have predominantly originated from development-phase or vendor-affiliated cohorts and have been assessed against curated case selections rather than against the unselected, heterogeneous case mix encountered in routine acute NCCT imaging [18]. Three methodological gaps remain particularly evident. First, prospective designs employing consecutive enrolment of clinically suspected stroke patients, as opposed to retrospective sampling from confirmed-stroke registries, are scarce, leaving unresolved the question of how the algorithm behaves when applied to the spectrum of presentations actually triaged through emergency NCCT, in which acute pathology constitutes the minority finding and pre-test probability is markedly heterogeneous. Second, evaluations conducted by investigators fully independent of the developer remain limited, although such independence is increasingly recognised as a precondition for unbiased performance estimation in medical artificial intelligence [12,14]. Third, granular sub-analyses, including regional sensitivity by anatomical location, behaviour of the continuous confidence score across correctly and incorrectly classified cases, robustness to imaging artefacts, and sensitivity to alternative operating thresholds, have not been systematically reported, notwithstanding their direct relevance to clinical interpretation and site-specific recalibration. The present study was designed to address these gaps by undertaking, to the best of the authors’ knowledge, one of the first prospective independent external validations of the BrainScan platform in an unselected, consecutively enrolled emergency cohort, with pre-specified regional, confidence-score, artefact, and threshold-sensitivity sub-analyses.

Addressing those gaps, our study contributes to the existing AI stroke validation literature. We represent an independent prospective evaluation in an unselected consecutive emergency cohort (rather than a curated case-confirmed sample), pre-specified granular sub-analyses spanning regional sensitivity, confidence-score behaviour, artefact robustness, and threshold sensitivity, and an explicit quantitative bias analysis bounding the impact of imperfect reference-standard ascertainment on the primary estimates.

### 1.4. Aim of the Study

The aim of this study is to assess the performance of an AI-driven stroke imaging interpretation tool in a cohort of patients presenting with acute ischemic stroke. We will compare the software’s analysis of patients’ initial brain scans with the interpretations by two independent expert radiologists. By directly comparing the AI’s detections to the radiologists’ readings, we seek to determine the software’s accuracy, sensitivity, and specificity in a real-world clinical setting. This head-to-head comparison will highlight both the strengths and any weaknesses of the AI tool. Our ultimate goal is to benchmark the performance of the AI system against expert radiologist interpretation in identifying acute stroke findings on imaging.

## 2. Materials and Methods

### 2.1. Study Design and Setting

This study was designed as a prospective, single-centre external validation study conducted at UMHAT “St. George”, Plovdiv, Bulgaria.

The system used in our study is BrainScan AI software (version 1.4; BrainScan sp. z o.o., Warsaw, Poland), which is a CE-marked, regulatory-approved commercial decision-support system for the automated analysis of non-contrast head CT. The system is designed for triage and prioritisation of acute intracranial pathology. The platform was developed independently by the manufacturer and acquired by the participating institution through standard procurement channels. The investigators had no involvement in algorithm development, training, validation, or selection of operating parameters, and have no financial or contractual relationship with the developer. The system itself is based on a deep convolutional neural network trained on a proprietary multi-institutional dataset. Specific architectural details, training data composition, augmentation strategy, hyperparameters, and internal calibration procedures are not disclosed by the manufacturer to end users and were also not available to the investigators.

For each NCCT examination, the platform returns a binary classification (positive/negative) and a continuous confidence score in the range [0, 1] for each of the target pathologies (ischemia and intracranial haemorrhage, with subtype stratification for the latter). No feature attribution, saliency map, internal layer activation, or other model-introspection output is exposed. The deployed binary classification operates at a manufacturer-defined default threshold whose numerical value, calibration population, and derivation rationale are not disclosed [18]. The implications of this opacity for the present analyses are addressed in the Section 4.

The study enrolled consecutive adult patients admitted on an emergency basis between January 2025 and December 2025 with a clinical suspicion of acute ischemic stroke. All included patients underwent NCCT as part of the standard acute stroke diagnostic protocol. It is acknowledged that early ischemic changes may be absent on initial NCCT. NCCT scans were independently interpreted by two board-certified radiologists with experience in neuroimaging, who were blinded to each other’s assessments and to the AI output. Each radiologist evaluated the scans for the presence of acute ischemia and intracranial hemorrhage.

In cases of disagreement between the two radiologists, a consensus reading was performed after joint review and discussion. Formal quantification of inter-rater reliability (e.g., Cohen’s κ) was not performed; the implications of this omission for reference standard validity are discussed in the Section 4. The reference standard for the diagnosis of acute ischemic stroke was defined using a composite clinical-radiological approach. This included: (i) initial NCCT interpretation by two independent radiologists with consensus adjudication in cases of disagreement; (ii) follow-up neuroimaging where available; and (iii) clinical evaluation at discharge by a neurologist. It is acknowledged that this composite reference standard is temporally heterogeneous: the AI output and index radiological readings were evaluated against a diagnosis that, in a subset of cases, was established only retrospectively through follow-up imaging or clinical course. This asymmetry means that sensitivity estimates for both the AI system and the radiologists reflect performance against an outcome that was not fully ascertainable at the time of the index scan. The proportion of cases in which the reference standard was determined solely from follow-up or clinical confirmation (as opposed to index NCCT alone) should be reported to allow readers to assess the extent of this limitation.

Patients with initially negative NCCT scans but with persistent focal neurological deficits and subsequent imaging or clinical confirmation of ischemic stroke were classified as true ischemic stroke cases. This composite reference standard was used for all diagnostic accuracy analyses.

The final study population comprised 1260 patients (1419 NCCT examinations), of whom 704 were male (49.6%) and 715 were female (50.4%).

### 2.2. Eligibility Criteria

Patients were eligible for inclusion if they met all of the following criteria: age 18 years or older; either sex; clinical suspicion of acute ischemic stroke at presentation; and availability of a technically adequate NCCT of the head performed at the time of admission.

Patients were excluded if any of the following conditions were present: known diagnosis of epilepsy; presence of intracranial neoplasm; pre-existing neurodegenerative disorder; poor image quality precluding reliable radiological interpretation; or withdrawal of informed consent. These exclusion criteria were applied to minimise confounding from conditions that may mimic or obscure ischemic changes on non-contrast CT.

### 2.3. Clinical and Imaging Data Collection

Clinical data were collected prospectively using a standardised data collection form. Information was obtained from a detailed medical history, neurological examination performed at admission, review of hospital medical records, official radiology reports, and automated analysis reports generated by the BrainScan AI software. All data were anonymised prior to analysis and stored in a secure institutional database accessible only to the study investigators.

### 2.4. Non-Contrast CT Acquisition Protocol

All patients underwent non-contrast head CT using standardised institutional head CT protocols on a multidetector CT scanner (Siemens Healthineers, Erlangen, Germany). The core acquisition parameters, tube voltage, tube current modulation, rotation time, pitch, detector collimation, and primary reconstruction slice thickness, remained unchanged throughout the twelve-month study period. Minor protocol variants were present, confined to reconstruction slice thickness for supplementary thin-slice series and to the SAFIRE iterative reconstruction strength level. These adjustments were applied uniformly at an institutional level rather than varying arbitrarily between individual examinations, and affected neither the 5 mm primary brain series used for AI analysis and diagnostic reporting nor the fundamental acquisition geometry. No scanner hardware changes, software upgrades affecting image reconstruction, or unscheduled maintenance events resulting in calibration adjustments occurred during the study period. The AI input to BrainScan consisted exclusively of the 5 mm primary brain series, which was produced under consistent acquisition and reconstruction conditions throughout.

Scans were acquired in helical mode from the skull base to the vertex with gantry tilt applied to reduce radiation exposure to the orbits. Acquisition parameters included automated tube voltage selection (100–120 kVp), automated tube current modulation (CareDose4D) with reference tube current in the range of approximately 250–440 mAs, rotation time of 1.0 s, pitch of 0.55, and detector collimation of 32 × 0.6 mm.

Images were reconstructed with a primary slice thickness of 5 mm for diagnostic interpretation. Additional thin-slice reconstructions of 1–2 mm were generated for multiplanar reconstructions and post-processing. Reconstructions were performed using standard soft tissue brain kernels and high-resolution bone kernels.

Iterative reconstruction (SAFIRE, Siemens Healthineers) was used in most examinations to reduce image noise and radiation dose. Images were reconstructed using a 512 × 512 matrix and standard brain window settings.

### 2.5. BrainScan AI Analysis

The BrainScan artificial intelligence platform was integrated into the hospital’s picture archiving and communication system (PACS) and processed DICOM datasets from NCCT imaging. Following image acquisition, scans were automatically transferred to the BrainScan platform for analysis without manual interaction by the investigators. The software provided automated binary classification of detected abnormalities as ischemic or haemorrhagic, together with a corresponding continuous confidence score (expressed as a probability) reflecting algorithmic certainty for each classification. The software version used throughout the study was version 1.4 (release date: 8 July 2025).

The reference standard used for comparison with the BrainScan AI output was the composite clinical-radiological diagnosis as defined in Section 2.1. Radiologists were blinded to the AI output at the time of reporting. The AI output was not available to treating clinicians and did not influence clinical decision-making. The reference standard diagnosis was used directly as the comparator for all diagnostic accuracy analyses; no additional adjudication procedure beyond the predefined consensus reading was applied. The inherent limitations of this reference standard definition, including the absence of formal inter-rater reliability quantification, are discussed in the Section 4.

### 2.6. Statistical Analysis

#### 2.6.1. Software and Descriptive Statistics

Statistical analysis was performed using R (version 4.5.1; R Foundation for Statistical Computing, Vienna, Austria) with the following packages: tidyverse (data management and visualisation), epiR (diagnostic accuracy metrics), pROC (ROC analysis), gtsummary (descriptive tables), ggplot2 (figures), and kkstatfun [19].

Continuous variables are reported as mean (standard deviation) when approximately normally distributed, supplemented by median and interquartile range. Categorical variables are reported as absolute frequencies and proportions.

#### 2.6.2. Primary Diagnostic Accuracy

The primary outcomes of the study were diagnostic accuracy for ischemia detection and diagnostic accuracy for hemorrhage detection, each evaluated against the composite reference standard and quantified by sensitivity, specificity, positive and negative predictive values, positive and negative likelihood ratios, and the area under the receiver operating characteristic curve. All remaining analyses, including threshold sensitivity analysis, regional breakdown of ischemia sensitivity, confidence score distribution, artefact impact, and McNemar direct comparison were designated as secondary or exploratory prior to data analysis and should be interpreted accordingly, without adjustment for multiplicity in the context of hypothesis generation.

For each condition, a 2 × 2 contingency table was constructed from the binary AI classification (positive/negative) and the reference standard, from which the following metrics were derived: sensitivity, specificity, positive predictive value (PPV), negative predictive value (NPV), positive likelihood ratio (LR+), negative likelihood ratio (LR−), diagnostic odds ratio (DOR), Youden index, and overall correctly classified proportion. All binary diagnostic metrics were computed using exact (Clopper–Pearson) 95% confidence intervals via the epiR package.

#### 2.6.3. ROC Analysis

Receiver operating characteristic (ROC) analysis was conducted using a composite continuous score variable constructed in three tiers. For AI-flagged scans (true-positive and false-positive cases), the above-threshold confidence score returned by the BrainScan platform was used directly. For false-negative cases in which the platform returned a sub-threshold confidence output, that sub-threshold value was used. For all remaining non-flagged scans: false-negative cases for which no sub-threshold output was available, and all true-negative cases, a score of zero was imputed, on the assumption that the absence of any output signal is appropriately represented by the minimum of the score range. The AUC was estimated by the DeLong method with 95% confidence intervals; the optimal operating threshold was identified by maximising the Youden index.

#### 2.6.4. Regional and Artefact Analyses

Regional diagnostic performance was assessed by computing sensitivity with Wilson score 95% confidence intervals for each anatomical region and hemisphere, restricted to scans in which the respective region was involved according to the reference standard. These Wilson score intervals differ from the exact Clopper–Pearson intervals used for the primary diagnostic accuracy metrics; estimates from regional and primary analyses should not be directly compared with respect to interval width. The effect of imaging artefacts on AI performance was evaluated using Fisher’s exact test, with the odds ratio and corresponding 95% confidence interval reported.

#### 2.6.5. Logistic Regression of Correct Classification

Predictors of correct AI classification among confirmed pathology cases were examined using binary logistic regression, with AI detection outcome (correct/incorrect) as the dependent variable. Continuous predictors were standardised prior to modelling. Given the very small number of false-positive events for both ischemia (*n* = 2) and hemorrhage (*n* = 2), false-positive models were not fitted; these cases are described descriptively. Convergence warnings arising from near-complete separation in the hemorrhage sensitivity model are acknowledged as a limitation of that analysis, and its results are reported with corresponding caution.

#### 2.6.6. Comparative Testing, Sensitivity Analyses, and Inferential Framing

The McNemar test for paired binary data was used to assess the significance of discordance between AI and radiologist classifications, and to compare AI classification accuracy between ischemia and hemorrhage detection.

Sample size adequacy was assessed post hoc using a precision-based framework. No prospective sample size calculation was performed because the study enrolled consecutive patients presenting under the routine acute stroke imaging protocol over a fixed twelve-month period, with the achieved sample size determined by clinical throughput rather than by an a priori inferential target.

A pre-specified sensitivity analysis was also conducted. To assess robustness to threshold choice, ischemia diagnostic accuracy was recomputed at six alternative confidence score thresholds ranging from 0.055 to 0.30, given that the Youden-optimal threshold was notably low.

A two-sided significance threshold of α = 0.05 was applied throughout. Accordingly, all subgroup, regional, and secondary analyses should be interpreted as hypothesis-generating. Results are reported with 95% confidence intervals to convey estimation precision; inferential language (e.g., “significant,” “no significant difference”) in these secondary analyses should be understood in the context of unadjusted testing across multiple comparisons, and no claim of confirmatory inference is intended beyond the primary diagnostic accuracy metrics.

#### 2.6.7. Quantitative Bias Analysis for an Imperfect Reference Standard

Given that formal inter-rater reliability could not be quantified, the analytical dataset retained only the post-consensus reference standard rather than the two independent reader streams: a quantitative bias analysis was conducted to assess the sensitivity of the primary ischemia accuracy estimates to plausible non-differential misclassification of the reference standard, using the closed-form 2 × 2 inversion approach for an imperfect reference standard [20,21]. Two complementary analyses were performed. A deterministic grid analysis recomputed the corrected sensitivity and specificity at fixed combinations of reference-standard sensitivity (Se_R in {0.95, 0.97, 0.99}) and specificity (Sp_R in {0.97, 0.99, 0.999}), yielding a bounded sensitivity envelope across plausible reference-standard parameters. A complementary probabilistic analysis treated Se_R and Sp_R as random variables drawn from Beta priors centred on values consistent with consensus radiologist reading for acute ischemia on NCCT (Se_R: Beta(92, 8), mean = 0.92; Sp_R: Beta(196, 4), mean = 0.98), with 50,000 Monte Carlo iterations. For each draw, the observed 2 × 2 contingency table was inverted in closed form to recover bias-corrected cell counts; draws yielding infeasible (negative) cell counts were discarded, with the proportion retained reported transparently as itself informative about the compatibility of the prior with the observed data.

### 2.7. Precision-Based Sample Size Assessment

Because the study enrolled consecutive patients under the routine institutional acute stroke imaging protocol over a fixed twelve-month period, no prospective sample size calculation was performed; the achieved sample size was determined by clinical throughput rather than by a pre-specified inferential target. The adequacy of the achieved sample was therefore assessed using a precision-based framework rather than a post hoc power calculation (Table A1). This choice reflects the methodological consensus that observed (post hoc) power is a deterministic transformation of the observed *p*-value, conveys no additional inferential content beyond the confidence interval already reported, and is misleading when used to interpret a non-significant or imprecise result [22,23]. The half-width of the 95% confidence interval was instead adopted as the precision metric, with pre-specified acceptability thresholds of ±0.05 for sensitivity, specificity, positive predictive value, and negative predictive value, and ±0.03 for the area under the receiver operating characteristic curve.

All primary diagnostic accuracy estimates met these thresholds with the exception of ischemia sensitivity, for which the observed Wilson half-width was 0.070 (95% CI: 0.521–0.661, *n* = 201 confirmed ischemia cases), narrowly exceeding the ±0.05 threshold. All remaining estimates met the pre-specified precision targets, including ischemia specificity (half-width 0.003), ischemia AUC (0.024), and all hemorrhage metrics (half-widths 0.037 for sensitivity, 0.003 for specificity, and 0.016 for AUC).

To quantify the precision–sample-size relationship explicitly, the number of confirmed ischemia cases that would have been required to achieve specified precision targets, together with the corresponding total NCCT cohort sizes at the observed ischemia prevalence of 14.2%, is summarised in Table A1. Achieving a Wilson half-width of ±0.05 around an expected sensitivity of 0.60 would have required 366 confirmed ischemia cases, corresponding to approximately 2578 total NCCT examinations at the observed prevalence; achieving ±0.03 would have required 1021 cases and approximately 7191 examinations. The achieved sample of 201 confirmed cases (1419 examinations) therefore falls short of the ±0.05 target by a factor of approximately 1.8 for ischemia cases. This shortfall is consistent with the observed half-width of 0.070, since precision scales approximately with the square root of sample size and the implied scaling factor of 1.35 would, in expectation, reduce the half-width from 0.070 to approximately 0.052 if the ±0.05 target sample had been achieved. The imprecision of the ischemia sensitivity estimate is acknowledged as a study limitation, and the modest excess of the half-width over the pre-specified ±0.05 threshold is reflected in the cautious interpretation of this estimate throughout the manuscript.

## 3. Results

### 3.1. Study Population

The analysis comprised 1419 NCCT examinations obtained from 1260 individual patients. The cohort had a mean age of 66.2 years (SD 15.4; range 19–97), with an almost equal sex distribution (49.6% male and 50.4% female).

**Figure 1:**
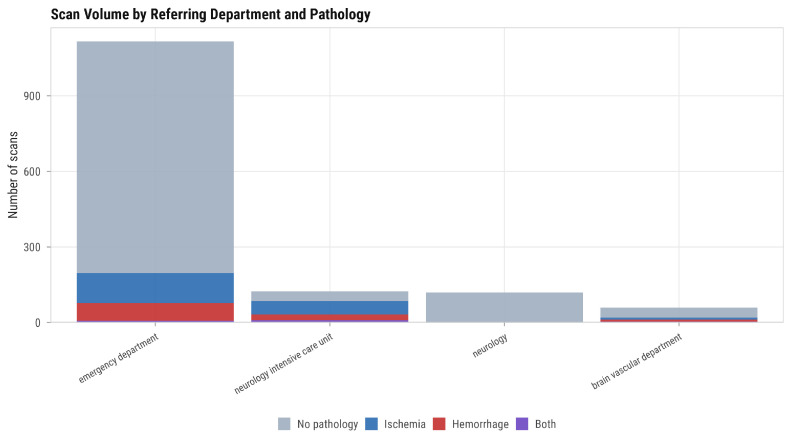
Distribution of NCCT examinations (*n* = 1419) by referring clinical department and diagnostic category. Diagnostic categories are derived from the composite reference standard (Section 2.1).

The majority of imaging studies originated from the emergency department, accounting for nearly four-fifths of all examinations, whereas substantially smaller proportions were referred from the neurology intensive care unit, the neurology department, and the brain vascular unit (Figure 1).

With respect to radiological findings, acute ischemic changes were identified in approximately one-seventh of scans (201 scans; 14.2%), while intracranial hemorrhage was present in a smaller subset (120 scans; 8.5%). A limited number of examinations demonstrated coexisting ischemic and hemorrhagic abnormalities (19 scans; 1.3%), whereas the large majority showed no evidence of acute intracranial pathology (1117 scans; 78.7%). Image quality was adequate in the majority of examinations. Artefacts were identified in 101 scans (7.1%), with artefact status missing for one scan; in most affected cases, artefact burden was partial rather than extensive, as reflected by a low median artefact proportion.

**Figure 2:**
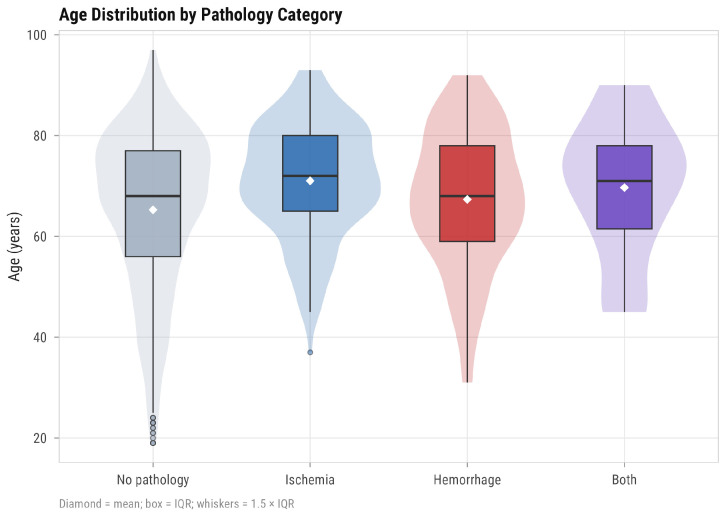
Distribution of patient age (years) across diagnostic categories defined by the composite reference standard. Box plots show median and interquartile range, with whiskers extending to 1.5 × IQR; individual observations beyond the whiskers are plotted. Denominators per category: no acute pathology *n* = 1117; ischemia *n* = 201; haemorrhage *n* = 120; combined ischemia and haemorrhage *n* = 19.

The distribution of age across pathology categories is illustrated in Figure 2, showing a tendency toward higher age in patients with vascular pathology compared to those without acute findings, while maintaining broadly comparable distributions between ischemic and hemorrhagic groups.

### 3.2. Ischemic Stroke Cohort

A total of 201 scans with radiologist-confirmed acute ischemia were included in the ischemic stroke cohort. Within this group, the BrainScan AI system identified ischemic changes in 119 cases, corresponding to a detection rate of 59.2%.

Among correctly identified cases, the AI-generated confidence scores were consistently high, with a median value of 0.79 (IQR 0.66–0.88), suggesting robust model certainty when ischemia was detected. In contrast, among missed cases, available sub-threshold confidence scores demonstrated a markedly lower distribution (median 0.18, IQR 0.00–0.35), indicating that undetected ischemic lesions were frequently assigned minimal probability by the algorithm.

From an anatomical perspective, ischemic lesions were predominantly located in the supratentorial compartment, with the highest regional frequencies observed in the basal ganglia, followed by the parietal, temporal, and frontal lobes. Lesion laterality was broadly symmetrical, with a comparable distribution between the left and right hemispheres, and a smaller subset of bilateral involvement.

### 3.3. Diagnostic Performance of BrainScan for Ischemia Detection

**Figure 3:**
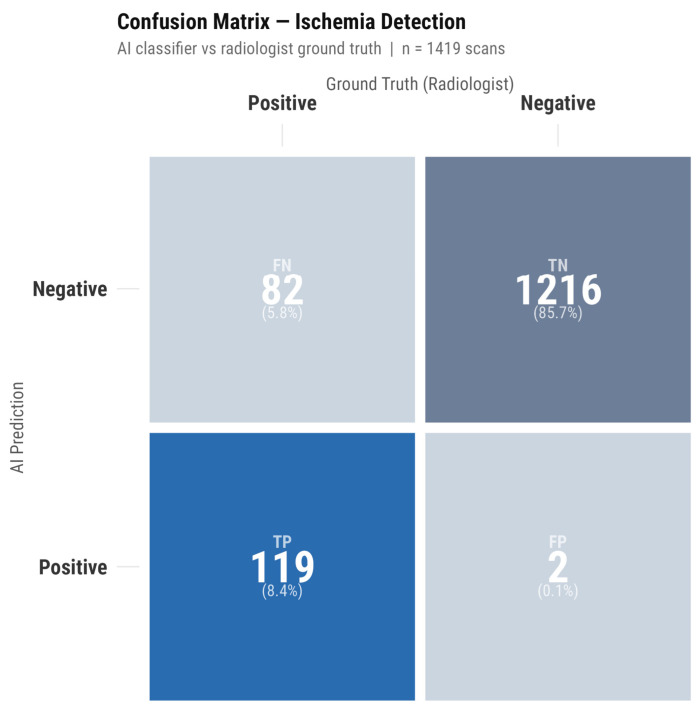
Confusion matrix comparing BrainScan AI binary classification with the composite reference standard for ischemia detection (*n* = 1419 NCCT examinations). Cell counts: true positive 119; false positive 2; false negative 82; true negative 1216. AI = artificial intelligence.

The diagnostic performance of the BrainScan AI system for the detection of acute ischemia is summarized in Table 1 and illustrated in Figure 3.

Overall, the AI model demonstrated a sensitivity of 59.2% (95% CI: 52.1–66.1%) and an exceptionally high specificity of 99.8% (95% CI: 99.4–100%). The positive predictive value (PPV) was 98.3% (119/121), while the negative predictive value (NPV) reached 93.7% (1216/1298). The near-absence of false-positive results further underscores the model’s high specificity.

#### Likelihood Ratios and Discordance

In terms of likelihood ratios, the positive likelihood ratio (LR+) was 360.6 (95% CI: 89.8–1446.9), indicating a very strong ability of the model to confirm ischemia when a positive result is generated. Conversely, the negative likelihood ratio (LR−) was 0.41 (95% CI: 0.35–0.48), reflecting a moderate ability to exclude ischemia in negative cases. The overall diagnostic odds ratio (DOR) was 882.3 (95% CI: 214.3–3633.2), and the Youden index reached 0.59 (95% CI: 0.52–0.66), indicating a moderate overall discriminatory performance.

The confusion matrix (Table 1) reveals a pronounced asymmetry in classification errors, with 82 false-negative cases compared to only 2 false-positive cases. This pattern was confirmed by McNemar’s test, which demonstrated a statistically significant discordance between AI and radiologist classifications (*p* < 0.001).

### 3.4. ROC Analysis for Ischemia Detection

**Figure 4:**
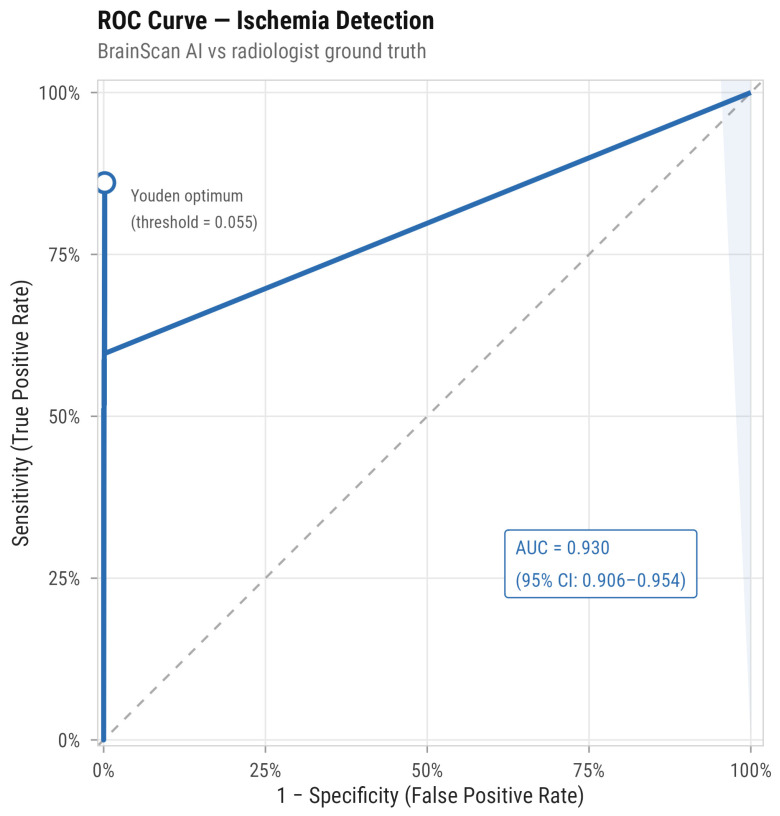
Receiver operating characteristic (ROC) curve for BrainScan AI ischemia detection (*n* = 1419), constructed from the AI-derived continuous confidence score with sub-threshold values incorporated for false-negative cases. Area under the curve (AUC) estimated by the DeLong method: 0.930 (95% CI: 0.906–0.954). The Youden-optimal threshold (0.055), corresponding to sensitivity 86.1% and specificity 99.8%, is indicated.

Using the three-tier composite score described in Section 2.6. Above-threshold scores for flagged cases, sub-threshold outputs for false-negative cases where available, and zero-imputed values otherwise, the ROC analysis yielded an AUC of 0.930 (95% CI: 0.906–0.954) (Figure 4). The AUC should be interpreted as conditional on the zero-imputation assumption for cases without sub-threshold output; the implications of this assumption are addressed in the Section 4. The optimal threshold determined by the Youden method was 0.055, yielding a sensitivity of 86.1% and a specificity of 99.8%.

The notably low Youden-optimal threshold of 0.055 warrants direct mechanistic interpretation. Three observations together account for it. First, the empirical distribution of confidence scores is markedly bimodal, with true-positive cases concentrated at high values (median 0.79, IQR 0.66–0.88) and true-negative cases concentrated near zero, separated by a wide low-density interval. Across this interval, sensitivity changes very little for a given incremental movement of the threshold, since few cases occupy intermediate score ranges; the Youden index is correspondingly flat across a broad threshold band, and the numerical optimum is determined by a small number of cases at the lower edge of the bimodal gap rather than by a sharply identified discriminating value. Second, the threshold-sensitivity analysis (Section 3.9) confirms this empirically: sensitivity is identical at thresholds of 0.055 and 0.10 (both 0.861), declines only modestly to 0.826 at 0.15, and falls progressively thereafter, indicating that the algorithm’s discriminative information is concentrated at the extremes of the score distribution rather than continuously distributed across the [0, 1] range. Third, the manufacturer’s default operating threshold, which is not disclosed but can be inferred to lie substantially above 0.055 from the observed binary classification performance (sensitivity 0.592 at default versus 0.861 at the Youden optimum), therefore reflects a deliberate calibration choice rather than an oversight: the developer appears to have selected an operating point that prioritises specificity at the expense of sensitivity, consistent with the high positive predictive value (98.3%) and very low false-positive rate (2 of 1218 reference-negative scans) observed in this cohort. The appropriateness of this default depends on the intended clinical use case and on the prevalence and case mix of the deployment population; the present results indicate that a moderate downward adjustment of the threshold could substantially recover sensitivity while preserving the specificity profile and support site-specific threshold recalibration as a routine component of clinical implementation. The numerical magnitude of the Youden-optimal threshold (0.055) should not, however, be interpreted as a recommended operating point in absolute terms, since the underlying confidence score is generated by a proprietary algorithm whose internal calibration is not transparent and whose values may not be directly comparable across software versions or deployment configurations.

### 3.5. Regional Analysis of Ischemic Detection

**Figure 5:**
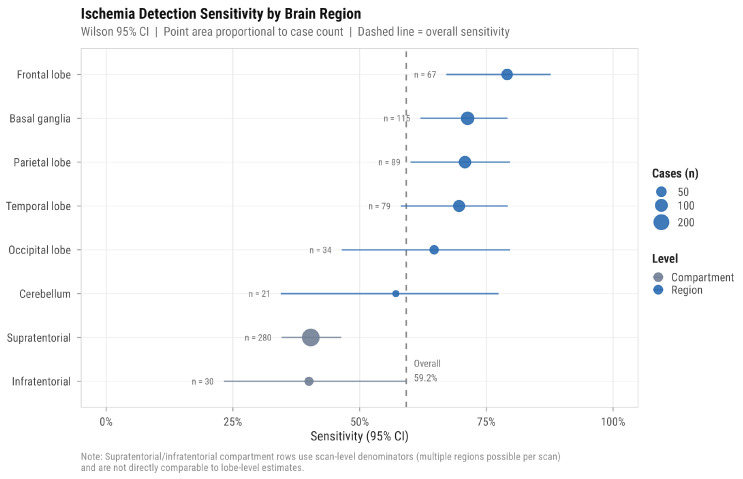
Regional sensitivity of BrainScan AI for ischemia detection across anatomical regions, with 95% Wilson score confidence intervals. Denominators are the number of region-level observations involving the respective region according to the reference standard. Lobe-specific and compartment-level rows differ in denominator definition and are not directly comparable.

The performance of the BrainScan AI system varied across different anatomical regions (Table 2, Figure 5). The highest sensitivity was observed in the frontal lobe (79.1%; 95% CI: 67.1–87.7%), followed by the basal ganglia (71.3%; 95% CI: 62.0–79.2%), parietal lobe (70.8%; 95% CI: 60.0–79.7%), and temporal lobe (69.6%; 95% CI: 58.1–79.2%). Lower detection rates were noted in the occipital lobe (64.7%; 95% CI: 46.5–79.7%) and the cerebellum (57.1%; 95% CI: 34.4–77.4%), while the lowest sensitivity was observed in infratentorial regions (40.0%; 95% CI: 23.2–59.2%).

The apparent discrepancy between supratentorial compartment sensitivity (40.4%) and the higher lobe-specific values (57.1–79.1%) reflects a difference in denominator definition rather than an inconsistency in performance. The compartment-level row counts each scan once if the respective compartment was involved, irrespective of which lobes contributed and whether multiple lobes were co-involved; an AI prediction is therefore counted as correct only if the system flagged the compartment as a whole.

Lobe-specific rows, in contrast, count region-level observations: a single multilobar scan contributes once to each involved lobe, and an AI flag in any one of those lobes counts as a correct detection for that lobe. Multilobar lesions, which are systematically more conspicuous and more likely to be detected, therefore inflate lobe-level sensitivities relative to the compartment-level figure. The two sets of estimates are not directly comparable and should be interpreted as addressing different questions: lobe-level sensitivity reflects the conditional probability of detection given involvement of a specific anatomical region, whereas compartment-level sensitivity reflects the unconditional probability of correctly flagging the compartment in any examination involving it.

Overall, these findings suggest that the AI system demonstrates improved sensitivity in cases with more extensive or anatomically conspicuous ischemic involvement, whereas performance is reduced in smaller or less distinct lesions, particularly within infratentorial regions.

### 3.6. Effect of Image Quality and Artifacts

The influence of image quality on AI performance was assessed by comparing scans with and without imaging artefacts. Among ischemia-positive cases, the presence of artefacts (*n* = 17) was associated with a sensitivity of 64.7% (95% CI: 38.3–85.8%), compared with 58.7% (95% CI: 51.2–65.9%) in artefact-free scans.

Statistical analysis using Fisher’s exact test did not demonstrate a statistically significant association between artefact presence and AI detection performance (OR = 1.29; 95% CI: 0.42–4.44; *p* = 0.80). This analysis was based on 17 artefact-positive ischemia cases and is substantially underpowered to detect a clinically meaningful difference; a null result should not be interpreted as evidence of the absence of an effect. However, this finding should be interpreted with caution given the relatively small number of ischemic cases with artefacts.

Importantly, specificity remained consistently high across both groups, reaching 99.9% in artefact-free scans and 98.8% in scans with artefacts, indicating that image quality had minimal impact on the model’s ability to correctly exclude ischemia.

### 3.7. Confidence Score Analysis

**Figure 6:**
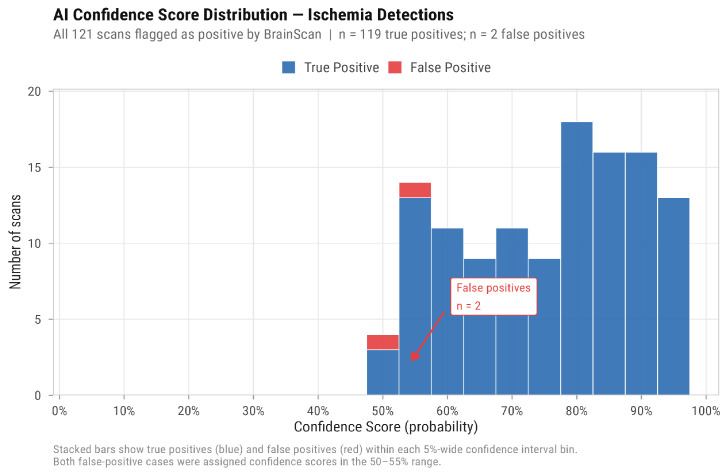
Distribution of BrainScan AI confidence scores for ischemia-positive predictions (*n* = 121), stratified by classification outcome against the reference standard. Bars represent true-positive cases (*n* = 119; median confidence score 0.79, IQR 0.66–0.88). False-positive cases (*n* = 2) are individually plotted as points above their respective bins with exact confidence score values. The x-axis displays the full probability range (0–100%); all AI-positive ischemia detections were assigned confidence scores of 50% or above.

The distribution of AI-derived confidence scores provided additional insight into model behaviour across correctly and incorrectly classified cases. The overall distribution of confidence scores for ischemia detections, stratified by true-positive and false-positive results, is presented in Figure 6.

As illustrated in Figure 6, correctly identified ischemic cases were associated with consistently higher confidence values, demonstrating a clear separation from false-positive detections. This pattern supports the internal consistency of the model’s probabilistic output and indicates that high-confidence predictions are highly reliable.

**Figure 7:**
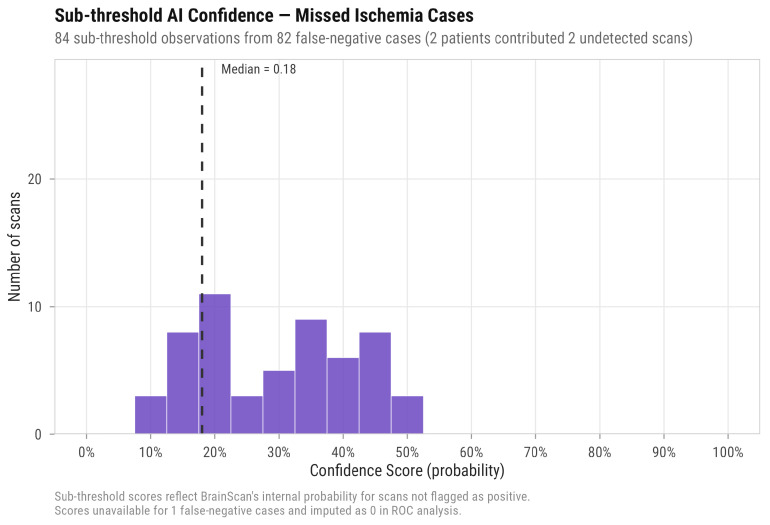
Distribution of sub-threshold BrainScan AI confidence scores among false-negative ischemia cases. Eighty-four sub-threshold observations were available from 82 false-negative cases (two patients contributed more than one undetected scan); all available observations are plotted. Median 0.18, IQR 0.00–0.35. The predominance of low values indicates that most missed cases reflect intrinsic model uncertainty rather than borderline classification errors near the operating threshold.

Further analysis of missed ischemia cases is shown in Figure 7, which depicts the distribution of sub-threshold confidence scores. Of the 82 false-negative cases, 84 sub-threshold score observations were available, as two patients contributed more than one undetected scan; the figure presents all available observations. In these cases, the confidence values were predominantly low, with a median of 0.18, indicating that the algorithm was generally uncertain rather than narrowly missing the classification threshold. This finding suggests that a substantial proportion of false-negative results correspond to intrinsically subtle or challenging imaging presentations.

Nevertheless, a subset of missed cases (approximately 25%) demonstrated intermediate confidence values (above 0.35), indicating that a modest reduction in the decision threshold could potentially recover additional true-positive detections. This observation is in line with the ROC analysis, where threshold optimization resulted in a marked increase in sensitivity while maintaining high specificity.

### 3.8. Comparative Performance: Ischemia vs. Hemorrhage

**Figure 8:**
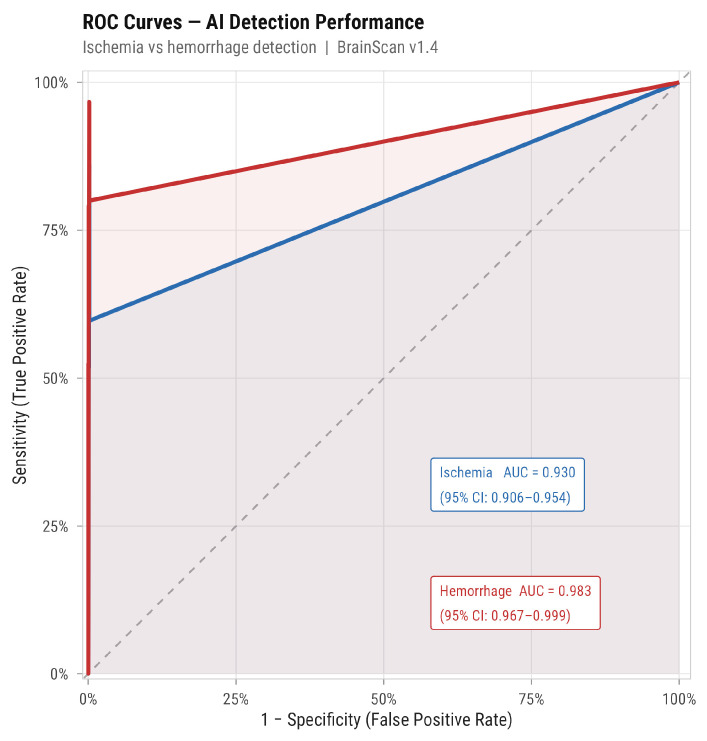
Receiver operating characteristic (ROC) curves comparing BrainScan AI performance for ischemia and haemorrhage detection (*n* = 1419 NCCT examinations). AUC: ischemia 0.930 (95% CI: 0.906–0.954); haemorrhage 0.983 (95% CI: 0.967–0.999).

A direct comparison of diagnostic performance between ischemia and hemorrhage is illustrated in Figure 8, which presents the combined ROC curves for both conditions. The model demonstrated superior discriminative ability for hemorrhage detection, with an AUC of 0.983, compared to 0.930 for ischemia.

As shown in Figure 8, the ROC curve for hemorrhage lies consistently above that for ischemia across the range of decision thresholds, indicating better overall sensitivity–specificity trade-off. This difference reflects the inherently higher detectability of hemorrhagic lesions on non-contrast CT compared to the often-subtle early ischemic changes.

A comprehensive summary of diagnostic metrics for both conditions is provided in Table 3. While specificity was equally high for both ischemia and hemorrhage (0.998), sensitivity differed markedly, with substantially higher values observed for hemorrhage (96.7%) compared to ischemia (59.2%). Similarly, negative predictive value and likelihood ratios further supported the superior performance of the model in hemorrhage detection.

#### Direct Comparison

Statistical comparison using McNemar’s test confirmed a significant difference in classification accuracy between the two conditions (χ^2^ = 67.4, *p* < 0.001), demonstrating that the AI system correctly identified hemorrhagic cases significantly more often than ischemic ones.

### 3.9. Sensitivity Analysis

**Figure 9:**
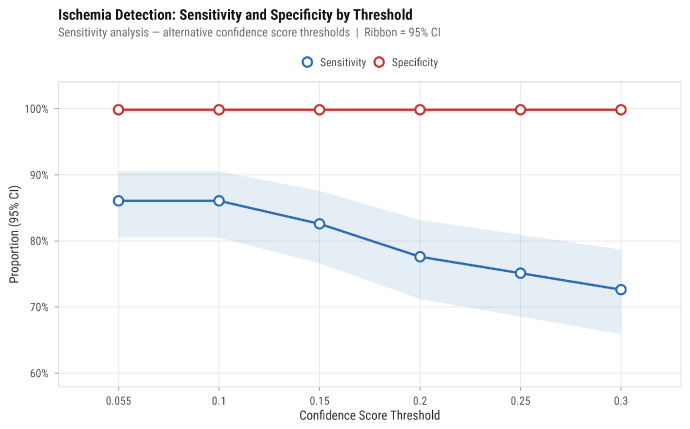
Sensitivity and specificity of BrainScan AI for ischemia detection across alternative confidence score thresholds (0.055, 0.10, 0.15, 0.20, 0.25, 0.30). Sensitivity is identical at thresholds 0.055 and 0.10 (0.861) and declines progressively thereafter; specificity remains fixed at 0.998 across all thresholds. Shaded bands represent 95% Wilson score confidence intervals. *n* = 1419 NCCT examinations; *n* = 201 reference-positive ischemia cases.

The BrainScan AI system assigns a continuous confidence score to each scan, with binary case flagging determined by an internal threshold. To assess the robustness of the primary ischemia detection results to threshold choice and given that the Youden-optimal threshold derived from the ROC analysis was notably low at 0.055, diagnostic accuracy was recomputed at five alternative thresholds ranging from 0.055 to 0.30. Specificity remained fixed at 0.998 (95% CI: 0.994–1.000) across all thresholds, confirming that the near-perfect specificity is a structural property of the algorithm independent of the operating threshold. Sensitivity was identical at thresholds of 0.055 and 0.10 (0.861, 95% CI: 0.805–0.905), indicating that no additional true-positive cases were captured in this score interval, consistent with a bimodal distribution of confidence scores around the detection boundary (Figure 9). At more conservative thresholds, sensitivity declined progressively: 0.826 (95% CI: 0.766–0.876) at 0.15, 0.776 (0.712–0.832) at 0.20, 0.751 (0.686–0.809) at 0.25, and 0.726 (0.659–0.787) at 0.30. These findings confirm that the primary binary detection performance is robust to modest upward threshold adjustments, though clinically meaningful reductions in sensitivity occur at thresholds exceeding 0.15. A second pre-specified sensitivity analysis was conducted to assess the influence of within-patient correlation arising from the inclusion of repeated scans. Of 1419 total examinations, 159 patients contributed more than one scan. Restricting the analysis to the index (first) scan per patient (*n* = 1260 unique patients, 138 ischemia-positive) yielded a sensitivity of 0.536 (95% CI: 0.449–0.621) and an AUC of 0.912 (95% CI: 0.881–0.944) for ischemia detection, compared with 0.592 and 0.930 in the full cohort, representing a difference of 5.6 percentage points in sensitivity. Specificity remained 0.998 (95% CI: 0.994–1.000). All point estimates and confidence intervals from the first-scan analysis fell within or overlapped those of the full-cohort primary analysis, supporting the robustness of the findings to within-patient correlation, though the modest downward shift in sensitivity warrants acknowledgement in the interpretation of the primary estimate.

### 3.10. Quantitative Bias Analysis: Sensitivity to Reference-Standard Misclassification

The deterministic grid analysis is summarised in Table 4. Of nine prior combinations, six were algebraically infeasible: any scenario with reference-standard sensitivity below 0.99 produced negative inverted cell counts and was therefore inconsistent with the observed contingency table. This is a substantive feature of the data rather than a numerical artefact: the algebraic feasibility constraint, applied to the observed near-absence of false positives (*n* = 2 in 1218 reference-negative scans), implies that the consensus reference standard must itself have sensitivity exceeding approximately 0.983. Within the feasible region (all combinations with Se_R = 0.99), the bias-corrected ischemia sensitivity ranged from 0.596 (when Sp_R = 0.999) to 0.728 (when Sp_R = 0.97), bracketing the observed estimate of 0.592 and lying within approximately 14 percentage points above it across the full feasible envelope. Bias-corrected specificity was essentially indistinguishable from the observed value across all feasible scenarios (0.999 in all three cases), reflecting the proximity of the observed estimate to the structural ceiling of the test.

The complementary probabilistic Monte Carlo analysis, performed under Beta priors with means of 0.92 (Se_R) and 0.98 (Sp_R), yielded a median corrected sensitivity of 0.656 (a shift of +0.064 relative to the observed) and a median corrected specificity of 1.000. Only 15 of the 50,000 iterations satisfied the feasibility constraint, mirroring the grid finding that the prior placed minimal mass in the region of parameter space compatible with the observed data; the prior-weighted simulation interval (0.607–0.776 for sensitivity) is therefore reported as exploratory, and the substantive interpretation rests on the bounded estimates of the deterministic grid. Across both analyses, the directionality of the correction, upward shift in sensitivity, and near-zero shift in specificity are consistent with established theory for non-differential misclassification of an imperfect reference standard, and the magnitude of the maximum plausible correction in sensitivity (approximately 14 percentage points at Sp_R = 0.97 and Se_R = 0.99) does not alter the substantive interpretation of the primary analysis. The corrected sensitivity remains well below that observed for hemorrhage detection in the same cohort and is consistent with the manuscript’s overall characterisation of the system as a rule-in tool for ischemia.

## 4. Discussion

This prospective external validation evaluated the real-world performance of the BrainScan AI system for ischemia detection on NCCT in a clinically representative cohort, addressing the well-recognised gap between development-phase and deployment-phase performance of medical AI systems [12].

The AI system demonstrated a characteristic performance profile with very high specificity (99.8%) and positive predictive value (98.3%), but only moderate sensitivity (59.2%). This asymmetry, reflected in the predominance of false-negative cases (*n* = 82 vs. *n* = 2 false positives), indicates a conservative classification strategy prioritizing specificity at the expense of sensitivity. From a clinical perspective, positive results are highly reliable, whereas negative results cannot safely exclude ischemia.

This performance pattern is consistent with prior literature. External validation studies have generally reported high specificity but more variable sensitivity for ischemia detection [14,24]. Murray et al. highlighted the substantial heterogeneity across AI models and evaluation methodologies, underscoring that generalizability remains a key challenge for AI-based stroke detection tools [11].

Variability in performance across datasets and clinical environments has been widely reported and remains a key barrier to generalizability in medical AI applications [12,15].

A central finding is the discrepancy between the model’s overall discriminative ability (AUC 0.930) and its binary classification performance at the default threshold (sensitivity 59.2%). Threshold optimisation through the Youden criterion substantially improved sensitivity to 86.1% while preserving the near-perfect specificity, indicating that the moderate sensitivity at the default operating point is predominantly threshold-driven rather than reflective of an intrinsic limitation of the underlying model [25,26]. The Youden-optimal threshold itself was notably low (0.055 on a [0, 1] scale), and the mechanistic basis for this finding is the markedly bimodal distribution of confidence scores observed in the cohort: true-positive cases were concentrated at high values (median 0.79) and true-negative cases at values near zero, separated by a wide low-density interval across which sensitivity is essentially insensitive to threshold choice. The threshold-sensitivity analysis confirmed this empirically, with sensitivity identical at 0.055 and 0.10 and declining only modestly to 0.826 at 0.15. The findings suggest that the manufacturer’s default operating threshold may prioritise specificity over sensitivity in routine deployment settings, consistent with the very high positive predictive value (98.3%) and the near-absence of false-positive classifications observed in this cohort. The appropriateness of this default depends on the intended clinical use case and on local prevalence and case mix, and the present results support site-specific threshold recalibration, rather than reliance on a manufacturer-defined default, as a routine component of clinical implementation [27]. The recalibrated threshold identified in the present cohort should be considered exploratory and hypothesis-generating and requires prospective validation in an independent cohort prior to operational implementation. The numerical magnitude of the Youden-optimal value should not be interpreted as a recommended operating point in absolute terms, since the underlying confidence score is generated by a proprietary algorithm whose internal calibration is not transparent and whose values may not be directly comparable across software versions or deployment configurations.

Confidence score analysis showed that correctly detected cases were associated with high probability values, whereas missed cases were predominantly assigned low confidence, indicating intrinsic model uncertainty rather than borderline classification errors. A subset of missed cases with intermediate confidence suggests that modest threshold adjustment could improve sensitivity.

The moderate sensitivity must be interpreted in the context of the intrinsic limitations of NCCT in early ischemia detection. Early ischemic changes are often subtle or absent on NCCT, which limits both human and algorithmic detection.

Regional analysis demonstrated higher sensitivity in supratentorial regions and reduced performance in infratentorial locations, consistent with known technical limitations of posterior fossa imaging. Higher detection rates in more extensive or bilateral lesions further suggest that model performance is influenced by lesion conspicuity.

The influence of image quality and artifacts did not significantly affect AI performance, although this finding should be interpreted cautiously given the limited number of artifact-positive cases. Specificity remained consistently high, suggesting robustness of the model in excluding ischemia even in the presence of moderate image degradation [28].

The AI system demonstrated substantially superior performance for hemorrhage detection, with higher sensitivity (96.7%) and AUC (0.983), consistent with prior studies. This performance gap reflects a fundamental difference in the physical basis of detection on non-contrast CT. Acute intracranial haemorrhage produces a marked increase in attenuation (typically 50–80 Hounsfield units, against a background of approximately 30–40 Hounsfield units for normal brain parenchyma), generating a high-contrast signal that is conspicuous to both human readers and convolutional neural networks within minutes of the bleeding event. Acute ischemia, by contrast, alters tissue attenuation by only a few Hounsfield units in the hyperacute phase, with detectable hypodensity emerging gradually over the first hours as cytotoxic oedema develops; up to one-third of acute ischemic strokes show no clear hypodense area on initial NCCT [5]. The signal-to-noise ratio available to any classifier, algorithmic or human, is therefore substantially lower for ischemia than for haemorrhage in the time window in which acute imaging is performed. This difference in physical conspicuity, rather than any architectural deficit specific to the AI system, accounts for the bulk of the observed performance gap and constrains the achievable sensitivity of NCCT-based ischemia detection irrespective of the classifier employed. The implication is that improvements in ischemia detection are likely to require integration with multimodal imaging that exploits more sensitive contrast mechanisms (CT perfusion, CT angiography, diffusion-weighted MRI), rather than further optimisation of NCCT-only classifiers in isolation.

Direct numerical benchmarking against comparable external validation studies clarifies where BrainScan stands within the field. For haemorrhage detection, the present sensitivity (96.7%) and AUC (0.983) are concordant with or exceed the benchmarks reported in pseudo-prospective and prospective external validations of comparable commercial platforms: VeriScout achieved sensitivity 0.92 (95% CI 0.84–0.96) and specificity 0.96 (95% CI 0.94–0.98) in an unselected emergency cohort [29]; Aidoc reported sensitivity 95.0% and accuracy 96.4% in a randomised reporting-time trial [30]; the Qure.ai algorithm reported AUC 0.92–0.94 in the CQ500 external validation [31]; and the pooled estimates from the most recent systematic review of AI haemorrhage detection on NCCT are sensitivity 0.90 and specificity 0.90 [32]. The present haemorrhage performance therefore sits at or above the upper end of published external benchmarks.

For ischemia, direct comparators on non-contrast CT are substantially scarcer than for haemorrhage: most published AI stroke validations target large vessel occlusion on CT angiography rather than parenchymal ischemia on NCCT, with reported LVO sensitivities of approximately 94% for proximal occlusions but 28–49% for distal M2 occlusions [33,34]. The reported BrainScan ischemia AUC of 0.930 is consistent with the upper range of NCCT-based ischemia detection performance described in the recent meta-analysis already cited [14], while the moderate sensitivity at the default operating threshold (59.2%) reflects the conservative manufacturer calibration described in Section 3.4 and Section 4 rather than a deficit relative to published external benchmarks at comparable operating points. Two interpretive points follow. First, the relative scarcity of NCCT-based ischemia external validation underscores the contribution of the present study to a thinner segment of the literature. Second, direct head-to-head comparison of BrainScan against alternative commercial systems on a common dataset, the only methodologically clean basis for ranking platforms, was beyond the scope of the present work and represents a clear priority for subsequent multicentre evaluation.

The defining performance characteristic of the system in this cohort: near-perfect specificity alongside moderate sensitivity at the default operating threshold, carries specific and asymmetric clinical implications that deserve explicit treatment. The near-absence of false positives (2 of 1218 reference-negative scans), reflected in the very high positive likelihood ratio (LR+ = 360.6), indicates that a positive BrainScan flag reliably identifies a true ischemic lesion and can support rapid escalation decisions, reperfusion pathway activation, urgent neurological review, or expedited transfer, with a very low risk of unnecessary intervention. Conversely, a negative result provides substantially weaker reassurance: 82 of 201 confirmed ischemia cases were not flagged at the default threshold, corresponding to a false-negative rate of 40.8%, an NPV of 93.7%, and a negative likelihood ratio of 0.41, the last indicating that a negative result is insufficient to meaningfully reduce the post-test probability of ischemia in patients with a clinically consistent presentation [35]. A negative BrainScan result in such patients cannot therefore be used to exclude ischemia or to defer further investigation, and clinical suspicion should override a negative flag. Predictive values are additionally prevalence-dependent, and the relatively low ischemia prevalence in the present cohort (14.2%) should be considered when extrapolating the NPV to populations with a higher pre-test probability. The system is appropriately positioned as a triage-support and rule-in adjunct, operating alongside clinical assessment and radiologist interpretation rather than as a replacement for either, consistent with conclusions drawn in recent evaluations of AI-assisted stroke triage systems [9,36]. Two operational consequences follow directly. First, local deployment should be accompanied by explicit institutional guidance that a negative AI result is not diagnostically conclusive for ischemia. Second, the threshold-sensitivity analysis (Section 3.9) demonstrates that a moderate downward adjustment of the operating threshold substantially recovers sensitivity (86.1% at threshold 0.055) with negligible loss of specificity, and site-specific threshold recalibration should be evaluated prospectively to determine whether a higher-sensitivity operating point is appropriate for the local case mix and clinical workflow, particularly in settings where the consequences of a missed ischemic lesion are especially severe, hyperacute reperfusion candidates, patients with posterior circulation or lacunar syndromes, and unaccompanied or poor-historian presentations in which clinical localisation is unreliable.

The operational corollary is that AI output should be integrated with, rather than substituted for, structured clinical evaluation. In the present cohort, the false-negative rate of 40.8% is bounded only by independent clinical signals: NIHSS-defined deficit profile, time of onset and last-known-well, vascular risk factor burden, and pre-test probability derived from focal neurological examination. A negative AI flag in a patient with an NIHSS-defined deficit, identifiable onset within the reperfusion window, and consistent vascular distribution should escalate rather than de-escalate the diagnostic pathway, with low-threshold progression to CT angiography, perfusion imaging, or diffusion-weighted MRI. Conversely, the very high positive likelihood ratio (LR+ = 360.6) means that a positive AI flag in a patient with a low pre-test probability, atypical presentation, alternative explanation for symptoms, and low vascular risk burden, should still prompt confirmatory imaging rather than direct activation of irreversible reperfusion pathways. The integrative model is therefore Bayesian rather than algorithmic: the AI output modifies but does not replace the post-test probability that follows from clinical assessment, and institutional deployment protocols should be designed around this combined decision logic rather than around AI thresholds applied in isolation. This positioning is also consistent with the rule-in framing developed earlier in this section and with the staged future-work agenda specified in the Conclusions, in which prospective evaluation of combined AI plus clinical decision rules is identified as a priority for subsequent multicentre work.

The characteristics of the study population further strengthen the relevance of these findings. The majority of scans originated from the emergency department, reflecting real-world clinical practice, and the age distribution across pathology groups was consistent with established epidemiological patterns of cerebrovascular disease. These factors enhance the external validity of the results and support their applicability to routine clinical settings.

This study has several strengths, including its prospective design, large sample size, inclusion of consecutive patients, and use of a composite reference standard. The analysis of confidence scores provides additional insight into model behavior beyond conventional binary metrics. Importantly, this represents the first independent prospective external validation of the BrainScan AI system in a clinically representative cohort, enhancing the generalizability of the findings beyond controlled development settings. Successful clinical implementation of AI systems requires not only high diagnostic accuracy but also appropriate integration into workflow, validation across diverse settings, and continuous performance monitoring [27].

Beyond the platform-specific findings, the methodological approach applied in the present study may help inform future independent external validation of comparable closed-source medical AI systems. Key elements include prospective consecutive enrolment, quantitative assessment of reference-standard uncertainty, pre-specified subgroup and threshold analyses, and transparent reporting of the limitations associated with proprietary algorithmic systems. The present findings should therefore be interpreted not only as an evaluation of BrainScan v1.4 in a real-world emergency cohort, but also as an example of how clinically deployed closed-source Neuro-AI platforms can be assessed under routine practice conditions.

Several limitations should be acknowledged:

First, the reference standard was derived from routine clinical radiological reporting rather than a prospective dual-reader protocol. The two independent reader streams were not retained in the analytical dataset; only the post-consensus diagnosis was recorded, and formal inter-rater reliability quantification (Cohen’s κ, percentage agreement) could not be reconstructed post hoc. A deterministic bias analysis under non-differential reference-standard misclassification was therefore conducted to bound the potential distortion of the primary estimates (Section 2.6, results in Section 3.10). Across the three algebraically feasible parameter combinations (reference-standard sensitivity 0.99; specificity 0.97, 0.99, and 0.999), bias-corrected ischemia sensitivity ranged from 0.596 to 0.728, and corrected specificity was 0.999 in all scenarios. The feasibility constraint itself is informative: scenarios with reference-standard sensitivity below 0.99 were algebraically incompatible with the observed contingency table, indicating that the near-absence of false positives implicitly imposes a strong lower bound on reference-standard accuracy. The headline conclusions are therefore robust to plausible reference-standard misclassification within the feasible parameter space. Future work will retain independent reader streams to permit direct κ quantification.

Second, no prospective sample size calculation was performed; consecutive enrolment under the routine stroke imaging protocol fixed the achievable sample by clinical throughput. Adequacy was assessed through a precision-based framework, as post hoc power calculation is mathematically equivalent to the observed *p*-value and provides no additional inferential content. All primary accuracy estimates met the pre-specified precision thresholds (95% CI half-width below 0.05 for sensitivity and specificity; below 0.03 for AUC) with the exception of ischemia sensitivity, for which the observed Wilson half-width was 0.070, narrowly exceeding the 0.05 target. Achieving that target would have required approximately 366 confirmed ischemia cases (roughly 2578 total NCCT examinations at the observed 14.2% prevalence), compared with the 201 cases achieved; under square-root-of-n scaling, this shortfall is consistent with the observed half-width and supports the call for prospective multicentre validation, in which a pooled denominator would deliver narrower precision around both the headline and regional sensitivity estimates.

Third, the study was conducted at a single tertiary university hospital using a single CT platform and standardised acquisition protocol. The performance estimates are therefore conditional on the acquisition, case-mix, and workflow characteristics of the participating centre. Three sources of between-centre variability that the present design cannot capture are acquisition-level differences (scanner manufacturer and generation, reconstruction kernel, iterative reconstruction algorithm, slice thickness), population-level differences (age and sex structure, prevalence and distribution of stroke aetiologies, lesion conspicuity, burden of chronic vascular change), and workflow-level differences (onset-to-imaging interval, referral mix, follow-up imaging practices, adjudication protocols). Any of these may shift sensitivity, specificity, or the optimal operating threshold relative to the values reported here. External replication across multiple centres and scanner platforms is required before these estimates can be considered broadly transportable. A formal temporal stability analysis evaluating whether AI performance varied systematically across the twelve-month enrolment period was not pre-specified and was not performed; future evaluations should address this through planned sub-period comparisons.

Fourth, 159 of the 1260 patients contributed more than one examination, yielding 1419 total scans. The inclusion of repeated scans introduces two distinct concerns: upward bias in point estimates from over-representation of multi-scan patients, and potential underestimation of standard errors from within-patient non-independence. The first concern is addressed by the pre-specified first-scan sensitivity analysis (*n* = 1260 unique patients; 138 ischemia-positive), which yielded ischemia sensitivity 0.536 (95% CI: 0.449–0.621) and AUC 0.912 (95% CI: 0.881–0.944), compared with 0.592 and 0.930 in the full cohort; the 5.6 percentage-point downward shift in sensitivity indicates modest upward inflation in the primary estimate. The second concern is addressed by a cluster-robust bootstrap resampling patients rather than scans across 1000 iterations, with all scans per sampled patient included in each replicate. The cluster-robust bootstrap interval for ischemia sensitivity was 0.519–0.660, compared with the scan-level Wilson interval of 0.521–0.661; the cluster-robust interval for specificity was 0.996–1.000, compared with 0.994–1.000. The near-identical width of the cluster-robust and scan-level intervals indicates that within-patient correlation does not materially affect the precision of the primary estimates. Future prospective evaluations should nevertheless pre-specify patient-level rather than scan-level as the primary unit of analysis.

Fifth, logistic regression models examining predictors of correct AI classification among confirmed ischemia cases produced extensive convergence warnings due to near-complete separation, a consequence of very few events in certain covariate strata. The hemorrhage sensitivity model was similarly affected by only four false-negative cases. Estimates from these models should be regarded as indicative rather than inferentially definitive.

Sixth, the study evaluated a single commercial AI platform (BrainScan, version 1.4), and findings cannot be extrapolated to other AI systems for stroke imaging, which vary in architecture, training data, operating thresholds, and intended use.

Seventh, the manufacturer’s default binary classification threshold is undisclosed; its derivation and calibration population were not available to the investigators. The Youden-optimal threshold derived from the present data (0.055) lies substantially below the manufacturer’s effective operating point, as evidenced by the gap between sensitivity at the empirical optimum (86.1%) and at the manufacturer’s default (59.2%). The mechanistic basis for this low value, the markedly bimodal score distribution and the wide low-density interval separating true-positive and true-negative cases, is described in Section 3.4 and Section 4. The opacity of the proprietary scoring logic limits interpretation of the absolute numerical magnitude of the threshold and precludes transport of threshold-related findings across software versions or deployment configurations.

Finally, BrainScan is a proprietary closed-source system; no feature attribution, saliency map, or other model-introspection output is accessible to end users, and no probing of the decision logic was undertaken or technically feasible. Two analytical consequences follow. The continuous confidence score is treated as an empirical signal whose statistical behaviour can be characterised but whose generation cannot be verified; whether it reflects a calibrated posterior probability or a vendor-specific transformation is unknown, and the absolute magnitude of any derived threshold is not interpretable in isolation or comparable across software versions. The composite score used in the ROC analysis assigned above-threshold confidence values to flagged cases, available sub-threshold outputs to false-negative cases where the platform returned them, and a zero-imputed value to all remaining non-flagged scans; the zero-imputation assumption is conservative and directionally appropriate, but the AUC should be interpreted as conditional on this assumption. These constraints are intrinsic to external evaluation of closed-source commercial AI and cannot be remedied by validation studies alone.

## 5. Conclusions

In this prospective external validation study, the AI system demonstrated excellent specificity and strong overall discriminative ability for ischemia detection on non-contrast CT, but only moderate sensitivity. The model showed a clear tendency toward conservative classification, resulting in highly reliable positive findings but a substantial proportion of missed ischemic cases.

Confidence score analysis showed that most false-negative results were associated with low model certainty, reflecting intrinsically subtle imaging features rather than borderline classification errors. Threshold optimization further indicated that sensitivity could be improved without compromising specificity, highlighting the importance of calibration in clinical deployment.

The system performed substantially better in hemorrhage detection than in ischemia, underscoring the inherent limitations of NCCT in identifying early ischemic changes. Regional variability further demonstrated reduced performance in infratentorial regions and improved detection in more extensive lesions.

Overall, these findings support the role of AI as a reliable decision-support tool in acute stroke imaging, particularly for confirming ischemia and prioritizing cases in high-throughput clinical settings. However, AI cannot replace expert radiological assessment and should be integrated as an adjunct rather than a substitute for clinical decision-making.

Before clinical implementation can be recommended, three sequential steps are required. First, prospective multicentre validation across institutions with heterogeneous scanner platforms, patient demographics, and imaging workflows is necessary to establish whether the performance profile observed here: high specificity, moderate sensitivity, and a conservative operating point, generalises beyond the present single-centre cohort; such a study should pre-specify patient-level as the primary unit of analysis and should retain independent reader streams to permit formal inter-rater reliability assessment. Second, site-specific threshold recalibration should be evaluated at each prospective deployment site, informed by the local ischemia prevalence and the clinical consequences of false-negative and false-positive results, given that the manufacturer’s default threshold was found to lie substantially above the Youden-optimal value derived in this cohort and that sensitivity is recoverable without specificity loss at a lower operating point. Third, integration with complementary imaging modalities, CT perfusion, CT angiography, and diffusion-weighted MRI should be explored to address the intrinsic limitation of NCCT for hyperacute ischemia detection, which constrains achievable sensitivity irrespective of classifier architecture. Pending this evidence, the system is appropriately deployed as a triage-support and rule-in adjunct under active radiological supervision, with explicit institutional guidance that a negative AI result does not exclude ischemia.

## Figures and Tables

**Table 1:** Confusion matrix for BrainScan AI ischemia detection against the composite reference standard (*n* = 1419 NCCT examinations). Cell counts: TP = 119; FP = 2; FN = 82; TN = 1216. TP = true positive; FP = false positive; FN = false negative; TN = true negative.

	Ground Truth +	Ground Truth −
AI Positive	TP = 119	FP = 2
AI Negative	FN = 82	TN = 1216

TP = true positive; TN = true negative; FP = false positive; FN = false negative.

**Table 2:** BrainScan AI sensitivity for ischemia detection by anatomical region, with 95% Wilson score confidence intervals (*n* = 201 reference-positive ischemia cases). Lobe-specific rows reflect region-level observations; supratentorial and infratentorial rows reflect compartment-level scan classification. The two are not directly comparable; see Section 3.5 for the denominator-definition distinction.

Region	*n*	Detected Cases	Sensitivity (95% CI)
Frontal lobe	67	53	79.1% (67.1–87.7%)
Basal ganglia	115	82	71.3% (62.0–79.2%)
Parietal lobe	89	63	70.8% (60.0–79.7%)
Temporal lobe	79	55	69.6% (58.1–79.2%)
Occipital lobe	34	22	64.7% (46.5–79.7%)
Cerebellum	21	12	57.1% (34.4–77.4%)
Supratentorial	280	113	40.4% (34.6–46.4%)
Infratentorial	30	12	40.0% (23.2–59.2%)

Values are presented as sensitivity with 95% confidence intervals. Supratentorial and infratentorial categories include cases where the respective region was involved, regardless of additional regions. *n* = number of region-level observations. Note: Supratentorial and infratentorial rows in Table 2 reflect scan-level classification across all examinations involving the respective compartment, irrespective of whether additional regions were co-involved. Lobe-specific rows reflect region-level analysis and are not directly comparable to compartment-level figures owing to differences in denominator definition. The apparent discrepancy between supratentorial compartment sensitivity (40.4%) and individual lobe sensitivities (57.1–79.1%) reflects this methodological distinction.

**Table 3:** Diagnostic performance of BrainScan AI for ischemia and haemorrhage detection against the composite reference standard (*n* = 1419 NCCT examinations). Values are point estimates with 95% confidence intervals (Clopper–Pearson for sensitivity, specificity, and predictive values; DeLong for AUC; standard formulae for likelihood ratios). PPV = positive predictive value; NPV = negative predictive value; LR+ = positive likelihood ratio; LR− = negative likelihood ratio; AUC = area under the receiver operating characteristic curve; Youden J = sensitivity + specificity − 1.

Metric	Ischemia (95% CI)	Hemorrhage (95% CI)
Sensitivity	0.592 (0.521–0.661)	0.967 (0.917–0.991)
Specificity	0.998 (0.994–1.000)	0.998 (0.994–1.000)
PPV	0.983 (119/121)	0.983 (116/118)
NPV	0.937 (1216/1298)	0.997 (1297/1301)
AUC	0.930 (0.906–0.954)	0.983 (0.967–0.999)
LR+	360.6 (89.8–1446.9)	627.9 (157.1–2508.7)
LR−	0.41 (0.35–0.48)	0.03 (0.01–0.09)
Youden J	0.590 (0.515–0.660)	0.965 (0.911–0.991)

Values are presented with 95% confidence intervals. PPV = positive predictive value; NPV = negative predictive value; LR+ = positive likelihood ratio; LR− = negative likelihood ratio; AUC = area under the curve.

**Table 4:** Bias-corrected ischemia sensitivity and specificity under fixed reference-standard parameters.

Reference-Standard Se_R_	Reference-Standard Sp_R_	Corrected Se	Corrected Sp
0.95	0.97	Infeasible *	infeasible
0.97	0.97	infeasible	infeasible
0.99	0.97	0.728	0.999
0.95	0.99	infeasible	infeasible
0.97	0.99	infeasible	infeasible
0.99	0.99	0.631	0.999
0.95	0.999	infeasible	infeasible
0.97	0.999	infeasible	infeasible
0.99	0.999	0.596	0.999

* “Infeasible” denotes parameter combinations that yield negative bias-corrected cell counts and are therefore inconsistent with the observed contingency table.

## Data Availability

The original contributions presented in this study are included in the article. The data supporting the findings of this study are available from the corresponding author upon reasonable request. However, due to privacy and ethical restrictions, raw patient data are not publicly available.

## Abbreviations

The following abbreviations are used in this manuscript:

CT	Computed Tomography
AI	Artificial Intelligence
NCCT	Non-Contrast Computed Tomography
ROC	Receiver Operating Characteristic
AUC	Area Under the Curve
CI	Confidence Interval
CTA	Computed Tomography Angiography
LVO	Large Vessel Occlusion
ASPECTS	Alberta Stroke Program Early CT Score
FDA	Food and Drug Administration
CE	Conformité Européenne
SAFIRE	Sinogram Affirmed Iterative Reconstruction
DICOM	Digital Imaging and Communications in Medicine
PACS	Picture Archiving and Communication System
PPV	Positive Predictive Value
NPV	Negative Predictive Value
LR+	Positive Likelihood Ratio
LR−	Negative Likelihood Ratio
DOR	Diagnostic Odds Ratio
ECASS	European Cooperative Acute Stroke Study
SD	Standard Deviation
IQR	Interquartile Range
OR	Odds Ratio

## Appendix A

**Table A1:** Required sample size for pre-specified precision of ischemia sensitivity.

Expected Sensitivity	Target 95% CI Half-Width	Required Ischemia Cases	Required Total NCCT Examinations
0.55	±0.05	377	2655
0.60	±0.05	366	2578
0.65	±0.05	347	2444
0.70	±0.05	320	2254
0.55	±0.04	591	4162
0.60	±0.04	573	4036
0.65	±0.04	543	3824
0.70	±0.04	502	3536
0.55	±0.03	1053	7416
0.60	±0.03	1021	7191
0.65	±0.03	968	6817
0.70	±0.03	894	6296

Required cases derived from the Wilson 95% confidence interval half-width formula. Required total NCCT examinations computed at the observed ischemia prevalence of 14.2% (rounded up to the nearest integer). Achieved in present study: 201 ischemia cases/1419 examinations; observed half-width 0.070.
